# ErbB3-Targeting Oncolytic Adenovirus Causes Potent Tumor Suppression by Induction of Apoptosis in Cancer Cells

**DOI:** 10.3390/ijms23137127

**Published:** 2022-06-27

**Authors:** Bo-Kyeong Jung, Young Jun Kim, JinWoo Hong, Han-Gyu Chang, A-Rum Yoon, Chae-Ok Yun

**Affiliations:** 1 Department of Bioengineering, College of Engineering, Hanyang University, 222 Wangsimni-ro, Seongdong-gu, Seoul 04763, Korea; yeesule@hanyang.ac.kr (B.-K.J.); mark95@hanyang.ac.kr (Y.J.K.); jhong803@gmail.com (J.H.); charley26@hanyang.ac.kr (H.-G.C.); ayoon@hanyang.ac.kr (A.-R.Y.); 2 GeneMedicine Co., Ltd., Seoul 04763, Korea; 3 Institute of Nano Science and Technology (INST), Hanyang University, 222 Wangsimni-ro, Seongdong-gu, Seoul 04763, Korea; 4 Hanyang Institute of Bioscience and Biotechnology (HY-IBB), Hanyang University, 222 Wangsimni-ro, Seongdong-gu, Seoul 04763, Korea

**Keywords:** oncolytic adenovirus, short hairpin RNA (shRNA), target, ErbB family, cancer cells, apoptosis, cancer gene therapy

## Abstract

Cancer is a multifactorial and deadly disease. Despite major advancements in cancer therapy in the last two decades, cancer incidence is on the rise and disease prognosis still remains poor. Furthermore, molecular mechanisms of cancer invasiveness, metastasis, and drug resistance remain largely elusive. Targeted cancer therapy involving the silencing of specific cancer-enriched proteins by small interfering RNA (siRNA) offers a powerful tool. However, its application in clinic is limited by the short half-life of siRNA and warrants the development of efficient and stable siRNA delivery systems. Oncolytic adenovirus-mediated therapy offers an attractive alternative to the chemical drugs that often suffer from innate and acquired drug resistance. In continuation to our reports on the development of oncolytic adenovirus-mediated delivery of shRNA, we report here the replication-incompetent (dAd/shErbB3) and replication-competent (oAd/shErbB3) oncolytic adenovirus systems that caused efficient and persistent targeting of ErbB3. We demonstrate that the E1A coded by oAd/shErbB, in contrast to dAd/shErbB, caused downregulation of ErbB2 and ErbB3, yielding stronger downregulation of the ErbB3-oncogenic signaling axis in in vitro models of lung and breast cancer. These results were validated by in vivo antitumor efficacy of dAd/shErbB3 and oAd/shErbB3.

## 1. Introduction

The ErbB family, an epidermal growth factor receptor (EGFR) family, is a transmembrane protein that consists of ErbB1, ErbB2, ErbB3, and ErbB4 (also known as human epidermal growth factor receptor (HER)1, HER2, HER3, and HER4, respectively). They function as homo- or heterodimers to activate downstream oncogenic signaling cascades [1]. Overexpression of ErbB family proteins and their mutations that result in constitutive activation of oncogenic signaling pathways are found in various types of human cancers with high frequency [2,3], making ErbB family proteins attractive targets for cancer therapy. Several preclinical and clinical trials have demonstrated that the antagonization of ErbB members (either ErbB1 or ErbB2 as main targets) with tyrosine kinase inhibitors (TKIs) such as gefitinib, erlotinib, or lapatinib, or monoclonal antibodies (Herceptin and Erbitux) provide promising components of an anticancer regimen [4]. 

The ErbB family-targeted therapy using one of the drugs mentioned above has been shown to elicit potent therapeutic effects in patients with solid tumors [5,6]. However, their efficacy diminishes over time due to *de novo* and acquired drug resistance [7,8,9,10]. In order to overcome this limitation, a combination of TKIs and monoclonal antibodies targeting either single or multiple receptors has been shown to enhance therapeutic efficacy in laboratory as well as clinical studies [11,12,13,14,15]. Nevertheless, a subset of patients still develop resistance to these complex regimens that cause a major hurdle in successful treatment [16]. The resistance to the ErbB family-targeted therapy can arise due to the following reasons: (1) mutation in the tyrosine kinase domain, resulting in enhanced kinase activity that promotes the dimer formation, (2) gene amplification, such as the ErbB family itself, and (3) activation of a compensatory pathway mediated by upregulation of ErbB3, c-Met, or Src family kinases [17,18,19,20]. Under these premises, the development of new treatment regimens that may overcome the drug resistance of conventional ErbB family-targeted therapeutic drugs is warranted. Since the interactions of ErbB3 with ErbB1 and ErbB2 are critical for oncogenic signaling, it has been anticipated that ErbB1- or ErbB2-overexpressing cancers could be promising targets [21,22]. ErbB3 is a kinase-deficient receptor, phosphorylated by dimerization with another ErbB family. In particular, dimerization of ErbB2 and ErbB3 is a preferred heterodimer formation that occurs with high frequency and these heterodimers function as the strong stimulator of the downstream oncogenic signal, particularly the phosphatidylinositol 3-kinase (PI3K) and protein kinase B (Akt) pathway, in breast and other solid tumors [23]. The phosphorylated ErbB3 (pErbB3) can bind directly to PI3K, a lipid kinase that promotes proliferation, survival, adhesion, and motility of cancer cells [24,25,26]. Additionally, it has been found that the overexpression of ErbB3 plays a critical role in transforming the activity of other ErbB family members in breast cancer cells [27]. Based on these reports, therapeutic strategies that block ErbB3 are currently being evaluated [28,29].

The RNA interference (RNAi) technique that offers sequence-specific degradation of messenger RNA has been successfully applied to various disease models including cancers [30]. Despite high specificity and efficacy, the application of small interfering RNA (siRNA) in clinic remains challenging due to its short half-life. Under these premises, developments of efficient siRNA delivery systems that overcome this limitation have been initiated [31]. One of these is a short hairpin RNA (shRNA)-expressing viral vector system, where vector-mediated expression of shRNA provides a prolonged and high level of RNAi expression in target tissues. Among several viral vectors that are currently being evaluated in various phases of clinical trials, an oncolytic adenovirus (Ad) is preferred because of its ability to preferentially replicate in and lyse cancer cells. Furthermore, it can be engineered to possess siRNA for proteins enriched specifically in a particular cancer cell type [32,33,34]. To this end, we previously demonstrated that the delivery of shRNA via the oncolytic Ad led to effective silencing of the target gene in cancer cells and reduction in tumor growth [35,36,37]. 

In the present study, we aimed to develop an Ad vector system expressing ErbB3-specific shRNA (shErbB3) for cancer therapy. We demonstrate that the Ad-mediated expression of shErbB3 could induce persistent silencing of ErbB3 and downregulation of its oncogenic signaling axis in in vitro models of breast cancer and that was well translated in in vivo tumor growth assays using a breast cancer xenograft model.

## 2. Results

### 2.1. ErbB3 Silencing by Replication-Incompetent Adenoviral Vector, dAd/shErbB3

**Figure 1:**
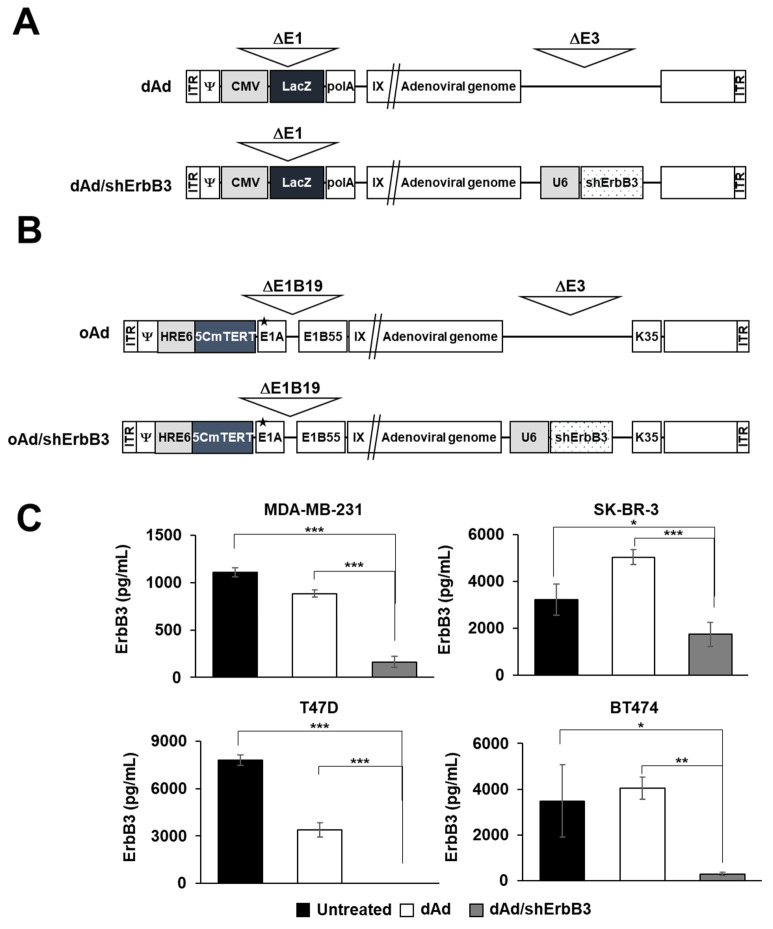
Schematic diagram showing Ad vector constructs used in the present study. (**A**) Replication-incompetent Ad (dAd) and replication-incompetent Ad-expressing shRNA against ErbB3 (dAd/shErbB3). (**B**) Oncolytic Ad whose replication is regulated by modified TERT promoter (oAd) and oAd-expressing shErbB3 (oAd/shErbB3). (**C**) ErbB3 expression level in various breast cancer cells (MDA-MB-231, SK-BR-3, T47D, and BT474) transduced by dAd or dAd/shErbB3. Cancer cells were transduced with dAd or dAd/shErbB3 at an MOI of 50 (MDA-MB-231), 100 (SK-BR-3), 200 (T47D), or 100 (BT474). ErbB3 concentration was quantified in the cell lysate 48 h post-transduction using the human ErbB3/HER3 ELISA. The results represent the mean ± SD (n = 3 per group). * *p* < 0.05, ** *p* < 0.01, *** *p* < 0.001.

In order to induce effective and long-term silencing of ErbB3, we generated a replication-incompetent Ad-expressing shErbB3 from the E3 region of Ad (dAd/shErbB3; Figure 1A). ErbB3 silencing efficacy of newly generated Ad was investigated in various breast cancer cell lines. As shown in Figure 1C, MDA-MB-231, SK-BR-3, T47D, and BT474 cells transduced with dAd/shErbB3 showed significant downregulation (93.17%, 98.14%, 100%, and 87.92%, respectively) of ErbB3 as compared to the untreated cells. Of note, dAd-transduced cells showed 20.17% and 56.84% reduction in MDA-MB-231 and T47D cells; SK-BR-3 and BT474 cells showed an increase.

### 2.2. ErbB3 Silencing Caused Attenuation of Cell Proliferation 

**Figure 2:**
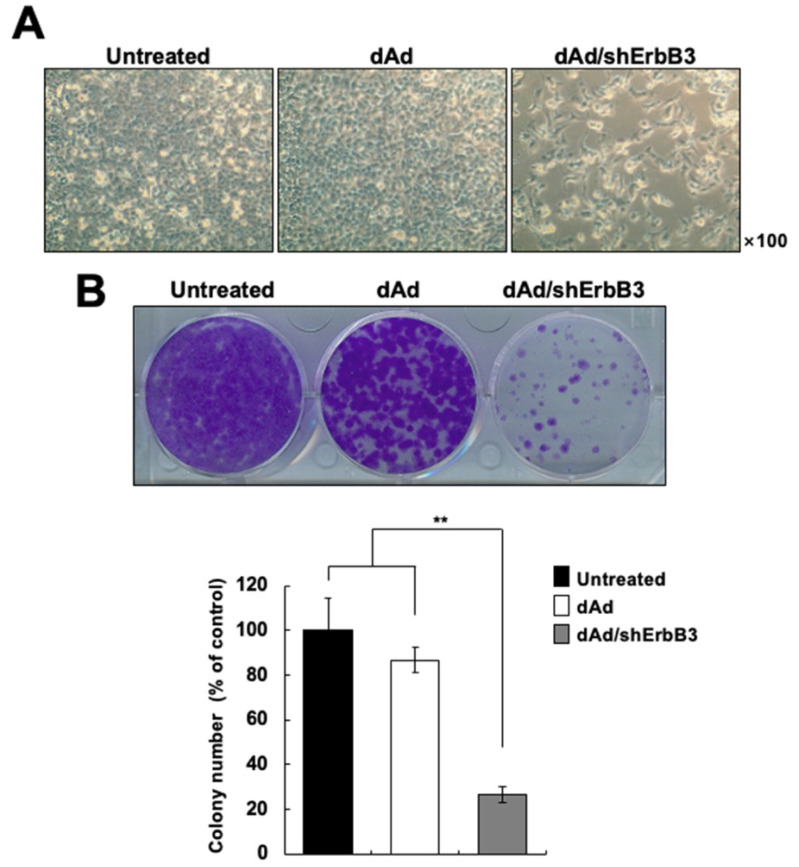
Cells transduced with Ad/shErbB3 showed decreased proliferation. (**A**) The representative photographs of cells transduced with either dAd or dAd/shErbB3 showing lower cell numbers in the latter. Magnification: ×100. (**B**) Representative image of colony forming assay. MDA-MB-231 cells were transduced with either dAd or dAd/shErbB3 at an MOI of 100. At 24 h post-transduction, the cells were detached and seeded in 6-well plates in triplicates at a density of 1 × 10^5^ cells/well. The photograph was taken after 14 days of incubation. The results represent the mean ± SD (n = 3 per group). ** *p* < 0.01.

The pErbB3 directly binds to PIK3, thereby contributing to cell proliferation and survival [38]. In light of this, we assessed whether Ad-mediated silencing of ErbB3 would lead to inhibition of cancer cell proliferation. MDA-MB-231 cells transduced with dAd or dAd/shErbB3 were observed under the microscope at 48 h after transduction. As shown in Figure 2A, the dAd/shErbB3-mediated silencing of ErbB3 led to a decrease in cell proliferation in comparison to those transduced with dAd. Furthermore, a colony-forming assay was performed to investigate the long-term effect of ErbB3 knockdown on the growth of cancer cells. At 14 days post-transduction, the cancer cells transduced with dAd/shErbB3 exhibited a significant reduction in colony number as compared to dAd-transduced cells (** *p* < 0.01; Figure 2B), indicating that the reduction of ErbB3 expression caused long-term inhibitory effects on anchorage-dependent growth of cells and colony formation.

### 2.3. ErbB3 Silencing Caused Induction of Apoptosis

**Figure 3:**
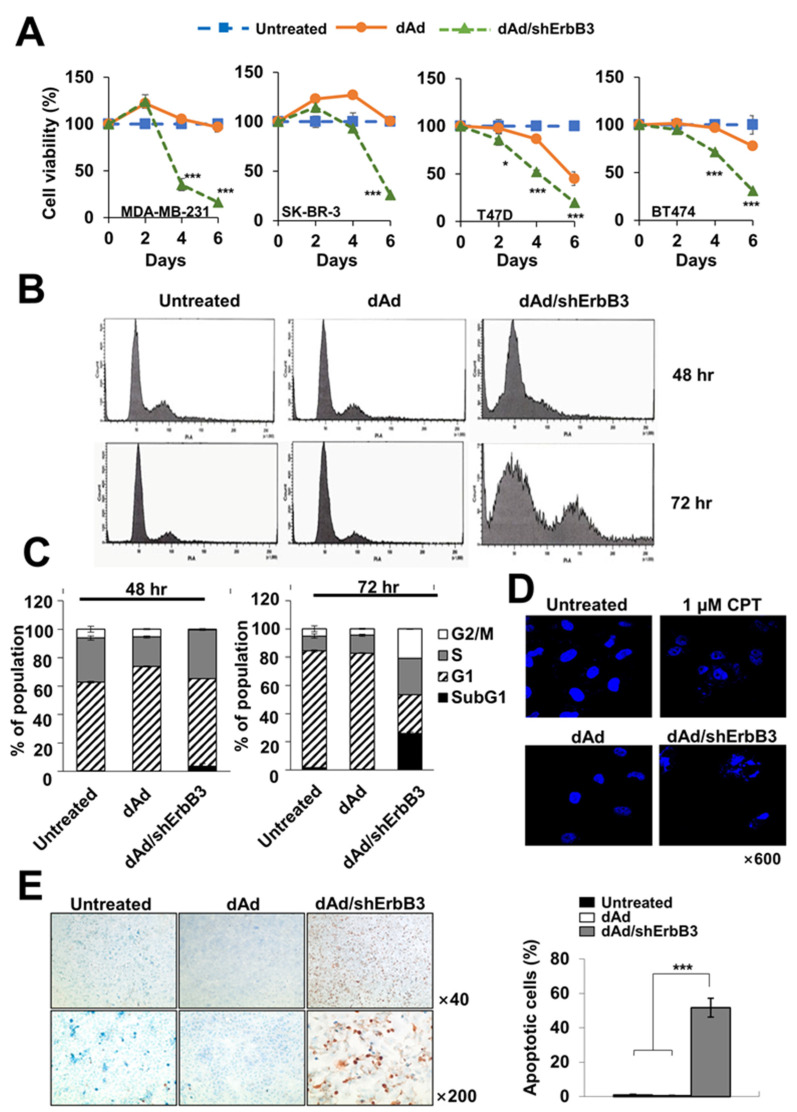
Induction of apoptosis by ErbB3 silencing. (**A**) Cells were transduced with either dAd or dAd/shErbB3 at various MOIs (MDA-MB-231, 200 MOI; SK-BR-3, 200 MOI; T47D, 50 MOI; BT474, 100 MOI) and cell killing efficacy was evaluated up to 6 days by MTT assay. The results represent the mean ± SD (n = 3 per group). * *p* < 0.05 or *** *p* < 0.001. (**B**,**C**) MDA-MB-231 cells were transduced with either dAd or dAd/shErbB3 (50 MOI). Cells collected 48 or 72 h post-transduction were stained with PI and analyzed by FACS. Results are expressed as a bar graph for the percentage of each phage of cell cycles. (**D**) MDA-MB-231 cells were transduced with 100 MOI of either dAd or dAd/shErbB3 then stained with Hoechst 33258 at 72 h post-treatment along with CPT as a positive control (magnification: ×600). (**E**) Representative images of TUNEL staining (magnification: ×40 and ×200) have been provided. The positive cells were counted and the result of the percentage of apoptotic cells was plotted as a bar graph. Data represent the mean ± SD (n = 4 per group). *** *p* < 0.001.

In order to investigate whether the expression of shErbB3 could induce a cancer cell killing effect, MTT assay was performed. As shown in Figure 3A, the cell viability was significantly lower in cancer cells transduced with dAd/shErbB3 as compared to those transduced with dAd in all the cell lines examined 6 days post-transduction (*** *p* < 0.001). Furthermore, we found that dAd/shErbB3 caused apoptosis (Figure 3B). As shown in Figure 3C, cell cycle analysis revealed a markedly higher population of cells in the subG1 phase in dAd/shErbB3-transduced cells than those transduced with dAd at 72 h after transduction. The number of cells in the G2/M phase was also higher in dAd/shErbB3-transduced cells than those transduced with dAd (4.52 ± 0.7% in dAd vs. 28 ± 1.3% in dAd/shErbB3), indicating a delay in mitosis. In line with these results, the microscopic observations of cancer cells stained with Hoechst revealed that the cells with either chromatin condensation or apoptotic bodies significantly increased in dAd/shErbB3-transduced cells (Figure 3D). Similar results were obtained by TUNEL staining wherein 51.7 ± 5.6% of the dAd/shErbB3-transduced cells showed apoptosis, whereas only 0.4 ± 0.4% of the dAd-transduced cells were apoptotic (Figure 3E, *** *p* < 0.001). Taken together, these results suggested that Ad-mediated silencing of ErbB3 caused cancer cell death by apoptosis.

### 2.4. Inhibition of Heregulin-Dependent and -Independent Cell Proliferation

**Figure 4:**
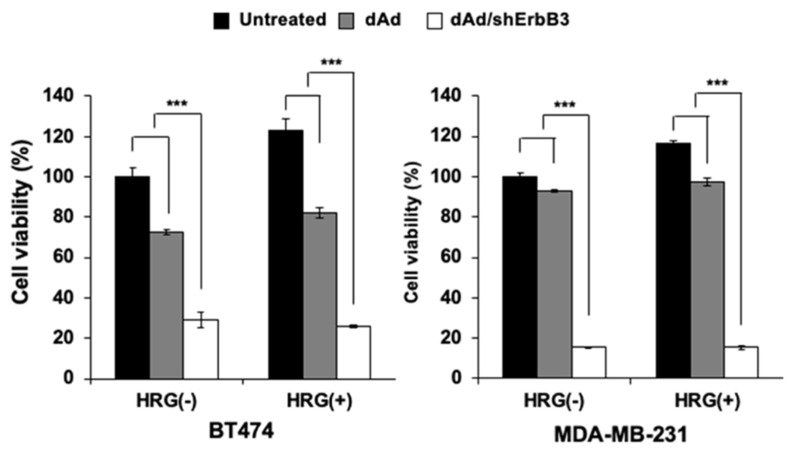
Inhibition of heregulin-dependent and -independent cell proliferation through ErbB3 downregulation. Cell viability was determined four days post-transduction of either dAd or dAd/shErbB3 into cells that were pre-treated with or without heregulin. Data represent mean ± SD (n = 3), *** *p* < 0.001.

Heregulin (HRG) is a specific ligand of ErbB3 [39,40]. The binding of ErbB3 with HRG induces the heterodimer formation of ErbB3 and other ErbB family members that, in turn, activates several signal transduction pathways including MAPK and PI3K/Akt signaling, resulting in cell proliferation, differentiation, and migration [41]. To examine whether shErbB3 could enhance the cell killing effect, ErbB2-overexpressing BT474 cells and ErbB2-underexpressing MDA-MB-231 cells were pre-treated with HRG (50 ng/mL) and transduced with dAd or dAd/shErbB. Then, 72 h post-transduction, cell viability was examined. As shown in Figure 4, cell viability of dAd/shErbB3-transduced cells decreased significantly in both ErbB2-high-expressing and ErbB2-low-expressing cells regardless of HRG treatment (*** *p* < 0.001 versus dAd).

### 2.5. dAd/shErbB3 Caused Potent Antitumor Effect

**Figure 5:**
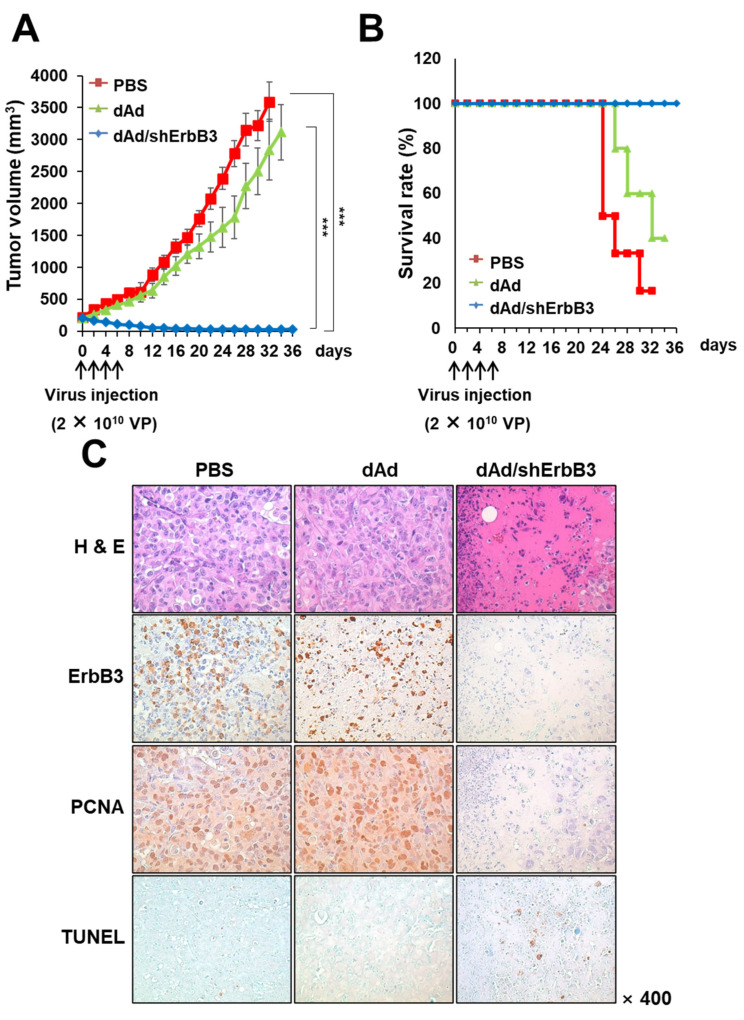
Tumor growth inhibition and increased survival rate of mice by ErbB3 silencing. Subcutaneous tumors derived from MDA-MB-231 cells were treated with 2 × 10^10^ VP of either dAd or dAd/shErbB3 along with PBS control (Q2D × 4) on Day 0, 2, 4, and 6. (**A**) Tumor volume was measured every other day. Data represented as mean ± SE (n = 5), *** *p* < 0.001. (**B**) Survival rate of mice treated with either PBS, dAd, or dAd/shErbB3 is shown. The percentage of surviving mice was determined by monitoring tumor volume growth-related events (tumor volume > 3000 mm^3^) over a period of 36 days. (**C**) Histological and immunohistochemical analysis. Tumors treated with PBS, dAd, or dAd/shErbB3 were harvested and stained with H&E or immunostained for ErbB3, PCNA, and TUNEL (magnification: ×400).

As demonstrated above, the shErbB3-expressing Ad caused remarkable decrease in breast cancer cell viability through the induction of apoptosis in vitro. In order to assess whether this effect would be retained in vivo, the antitumor effect of dAd/ErbB3 was examined in an MDA-MB-231 human breast cancer xenograft model. Subcutaneously implanted tumors were intratumorally injected with either PBS, dAd, or dAd/shErbB3 at a dose of 2 × 10^10^ VP every other day for a total of four times. As shown in Figure 5A, administration of dAd/shErbB3 significantly inhibited the tumor growth in mice compared with the dAd or PBS control (*** *p* < 0.001). PBS- and dAd-treated tumors increased to an average size of 3592.54 ± 304.52 mm^3^ and 2841.96 ± 475.54 mm^3^, respectively, by 32 days after the initial treatment. Of note, the average tumor volume of dAd/shErbB3-treated tumors was 30.53 ± 16.18 mm^3^, showing 99.15% growth inhibition in respect to the dAd-treated group, and two out of five mice in the dAd/shErbB3 treatment group showed complete tumor regression. In line with these results, the Kaplan–Meier survival data analysis revealed that the survival rate was significantly improved in tumor-bearing mice treated with dAd/ErbB3 compared with the dAd-treated group (Figure 5B). By 32 days following initial treatment, 100% of the animals in the dAd/ErbB3 group were still viable compared to only 40% in the dAd-treated group (*** *p* < 0.001, versus dAd-treated group). These in vivo data demonstrated that the adenovirus-mediated silencing of ErbB3 caused efficient tumor growth suppression, resulting in increased survival of the animals.

In order to verify the mechanism of enhanced antitumor effect and survival benefits of ErbB3 downregulation via the Ad vector, tumor tissues were excised and subjected to histological staining. As shown in Figure 5C, hematoxylin and eosin (H&E) staining revealed that the majority of the remaining tumor mass treated with dAd/shErbB3 was necrotic; such lesions were barely detectable in the tumors treated with dAd. To determine whether the enhanced antitumor effect of dAd/shErbB3-treated tumors coincides with reduced expression of ErbB3, tumor section slides were immunostained with the ErbB3-specific antibody. A marked decrease in ErbB3 expression was detected in the dAd/shErbB3-treated tumors compared with PBS- or dAd-treated tumor tissues. Further, PCNA expression was markedly reduced in tumors treated with dAd/shErbB3, suggesting that the proliferation of breast tumor cells was attenuated similar to those observed in vitro. Tumors treated with dAd/shErbB3 also showed the highest counts of TUNEL-positive spots in the necrotic area, further suggesting that the downregulation of ErbB3 also leads to apoptotic cell death in vivo.

### 2.6. ErbB3 Silencing by Replication-Competent Oncolytic Ad, oAd/shErbB3

**Figure 6:**
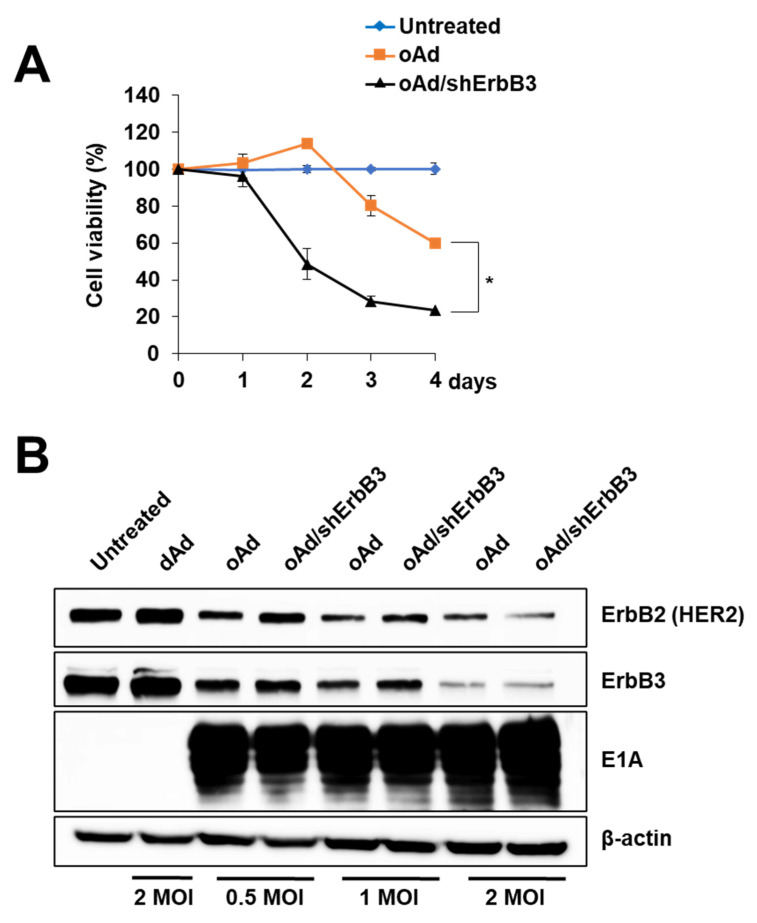
Downregulation of ErbB3 by oAd/shErbB3. (**A**) BT474 cells were infected with 2 MOI of oAd or oAd/shErbB3. The cell killing efficacy was evaluated for 4 days, followed by MTT assay. The results represent the mean ± SD (n = 3 per group). * *p* < 0.05. (**B**) BT474 cells treated with 0.5 to 2 MOI of oAd or oAd/shErbB3 were subjected to Western blot assay to verify ErbB2, ErbB3, and E1A expression.

In order to enhance and prolong the ErbB3 silencing effect mediated by Ad, we next constructed shErbB3-expressing oncolytic Ad, oAd/shErbB3, with knob substitution of Ad35 (Figure 1B). First, it was confirmed that the substitution of Ad35 to the knob improved intracellular transduction efficiency of Ad compared to the wild-type knob of Ad5 in various breast cancer cell lines (Figure S1). Further, to determine whether shErbB3 expression via the oncolytic vector can also lead to an enhanced cancer cell killing effect, an MTT assay was carried out. As shown in Figure 6A, the cell viability of oAd/shErbB3-infected BT474 cells significantly decreased by 76.66% by 4 days post-infection (* *p* < 0.01 versus oAd). Of note, although oncolytic Ad (oAd/shErbB3) was infected with a 50-fold lower dose compared to the replication-defective Ad (dAd/shErbB3) (Figure 3A), it induced a 2.54-fold higher cell killing effect in the same cell line; the cell viability of the dAd/shErbB3 vs. oAd/shErbB3 group on day 2—84.87% vs. 48.50% and day 4—71.72% vs. 28.27%, respectively. It was confirmed that the expression of shErbB3 did not interfere with viral replication (Figure S2). These results suggested that the shErbB3-expressing oncolytic Ad possessed enhanced ErbB3 silencing activity and further augmented the cancer cell killing effect of oncolytic Ad.

Several reports have suggested that the adenovirus E1A protein downregulates ErbB2 expression, which can possibly induce apoptosis [42,43]. To confirm whether the E1A protein reduces the expression of the ErbB2 protein, the ErbB2-overexpressing breast cancer cell, BT474, was infected with oAd or oAd/shErbB3 at MOI of 0.5–2; then, the expressions of E1A and ErbB2 were measured by Western blotting. As shown in Figure 6B, ErbB2 and ErbB3 expressions were attenuated in a dose-dependent manner by oAd and oAd/shErbB3, whereas the expressions remained unchanged by dAd, which does not express the E1A protein. Consistent with the previous findings, these data demonstrated that the oncolytic Ads suppress ErbB3 expression through a preserved E1A region. Taken together, these results showed that the simultaneous expression of both the Ad E1A region and shErbB3 by single oncolytic Ad can reduce both ErbB2 and ErbB3 expression, yielding higher anticancer efficacy.

### 2.7. oAd/shErbB3 Showed Stronger Antitumor Activity

**Figure 7:**
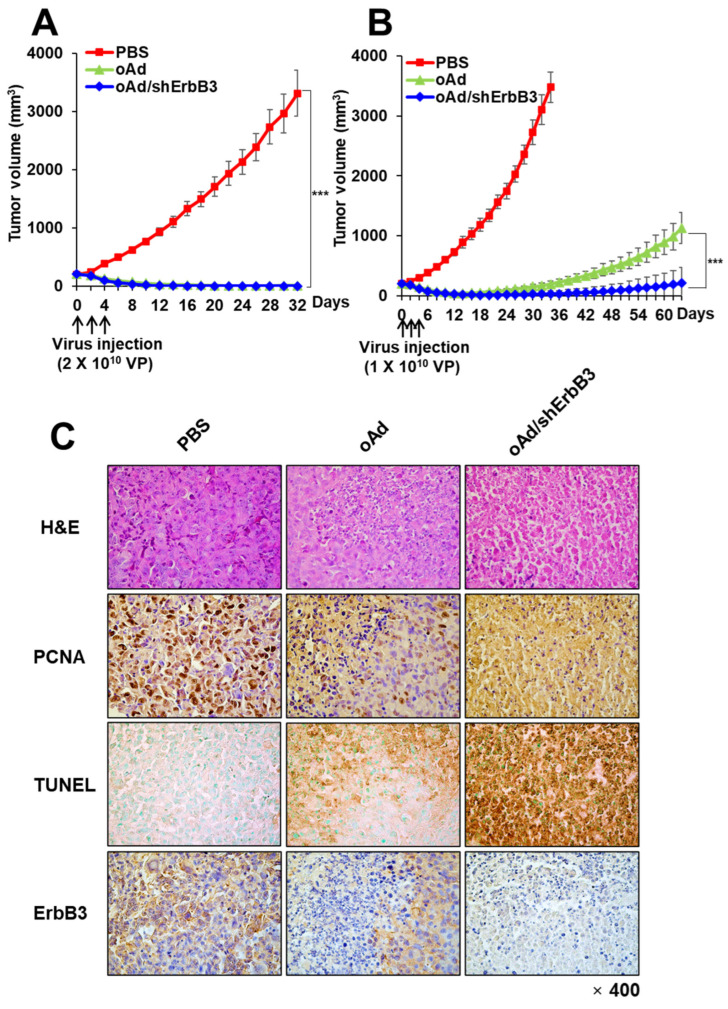
Tumor growth inhibition by shErbB3-expressing oncolytic Ad. Subcutaneous MDA-MB-231 tumor-bearing mice were injected with two different doses (1 × 10^10^ or 2 × 10^10^ VP) of oAd or oAd/shErbB3 along with PBS control (Q2D×3). (**A**,**B**) Tumor volume was measured every other day. Data represent as mean ± SE (n = 6), *** *p* < 0.001. (**C**) Histological and immunohistochemical analysis. MDA-MB-231 tumors treated with PBS, oAd, or oAd/shErbB3 (1 × 10^10^ VP, Q2D × 3) were harvested on Day 7. The tumor tissues were subjected to H&E staining and immunohistochemical staining for PCNA, TUNEL, and ErbB3 (magnification: ×400).

To assess the antitumor efficacy of oAd/shErbB3 in human breast cancer xenograft models, MDA-MB-231 tumors were subcutaneously established. As shown in Figure 7A, PBS treatment led to rapid growth of MDA-MB-231 tumors as the tumor volume reached 3312.42 ± 392.42 mm^3^ on day 32. Both oAd and oAd/shErbB3 (2 × 10^10^ VP, Q2D × 3) treatments induced significant tumor growth inhibition compared to PBS treatment (*** *p* < 0.001), resulting in complete regression of all tumors. Even at a lower dose of 1 × 10^10^ VP (Q2D × 3), both oAd and oAd/shErbB3 induced significant tumor growth inhibition compared to PBS treatment (Figure 7B; *** *p* < 0.001). Importantly, 1 × 10^10^ VP of oAd/shErbB3 (Q2D × 3) led to complete tumor regression in 50% of the mice and significantly more potent antitumor efficacy than oAd that persisted up to day 64 (*** *p* < 0.001): only one of the mice in the oAd group achieved complete tumor regression. Similar results were observed in the MCF-7/Mot breast cancer xenograft model where oAd/shErbB3 treatment induced significant reduction in tumor volume in respect to PBS or oAd treatment groups (Figure S3A; *** *p* < 0.001 or * *p* < 0.05, respectively). These data demonstrated that the suppression of ErbB3 expression by oncolytic adenovirus had strong inhibitory effects on tumor growth that was consistent with the in vitro data.

In order to evaluate the mechanism behind potent antitumor efficacy of oAd/shErbB3, histological analysis was performed. H&E staining revealed that the majority of the remaining tumor mass treated with oAd/shErbB3 was necrotic, whereas necrotic lesions were only detectable in the limited region of tumors treated with oAd (Figure 7B, Figure S3B). Similar to our in vivo results using dAd/shErbB3, oAd/shErbB3-treated breast tumors exhibited a markedly lower number of PCNA-positive cells, higher number of TUNEL-positive cells, and decreased level of ErbB3 compared to tumors that were treated with PBS or oAd. Together, these results demonstrate that oAd/shErbB3 exerted potent antitumor efficacy via robust inhibition of ErbB3 expression and inhibition of tumor cell proliferation, and enhanced induction of apoptosis in the tumor tissues.

## 3. Discussion

Targeting of activated oncogenic coding and noncoding pathways by RNA interference (RNAi) is considered as the most powerful, specific therapeutic strategy in treatment of genetic disorders including cancers. However, the major limitation of RNAi is that it is difficult to efficiently deliver them to target tissues and has a possibility of an off-target effect [44]. Accordingly, a variety of new viral and nonviral delivery systems have been introduced for improving the cellular uptake efficiency and reducing the off-target effects of RNAi [45].

Among the wide range of viruses (adenoviruses, alphaviruses, herpes simplex viruses, lentiviruses, retroviruses), adenoviruses have been used in preclinical and clinical cancer studies due to their safety, broad host cell permeability, high transduction efficiency, tumor selectivity, non-invasiveness, high genetic modifiability, and expression of transgenes [46,47,48]. Although replication-deficient adenoviruses had been more frequently investigated as a gene therapeutic for treating diseases other than cancer in the past [49,50], a majority of the recent clinical investigations of adenovirus in cancer therapy applications have employed oncolytic adenoviruses due to their distinct advantage of cancer-specific viral replication and cytolytic effect, leading to an increased transgene expression level and induction of antitumor immune response [51,52,53,54]. Due to these reasons, an oncolytic adenovirus causing RNAi can be highly advantageous in cancer therapy over the replication-incompetent counterpart, since shRNA can be expressed at a higher level with greater persistence in tumor tissues due to cancer-specific replication of the oncolytic virus and cascading infection of neighboring tumor cells. 

The ErbB oncogenic receptor tyrosine kinase family proteins (ErbB1, ErbB2, ErbB3, and ErbB4) have been shown to contribute significantly to pro-proliferation, migration, invasion, and drug resistance characteristics of diverse cancer cell types [55,56,57]. Several in vitro and in vivo studies have shown that ErbB3 acts as a key player in activating EGFR signaling and heterodimerization of ErbB2 and ErbB3 has been reported to induce resistance to ErbB2-targeted therapy [58,59]. Nrdp1 was shown to inhibit ErbB3 phosphorylation in colorectal cancer cells and suppress EGFR-MMP7 signaling-mediated metastasis [60]. Overexpression of miR-497, which targets Nrdp1 in human CRC cells, caused significant increase in MMP7 and metastatic properties of cells [60]. A variety of missense mutations in ErbB kinases have also been observed clinically and are related to drug resistance and poor therapeutic outcomes [61,62]. However, ErbB3-targeted therapies have not been so rewarded due to the complex biology of the receptor and multifactorial nature of carcinogenesis, as well as drug resistance [63,64]. Here, we generated an ErbB3-targeting adenovirus system with replication-incompetent and replication-competent oncolytic adenovirus. Both the systems caused efficient and persistent silencing of ErbB3 as marked by downregulation of its oncogenic signaling axis in in vitro breast cancer cells. Furthermore, by in vitro and in vivo assays, we found that the replication-competent oncolytic adenovirus caused E1A-mediated simultaneous downregulation for ErbB2 and ErbB3 proteins, accounting for its enhanced potency. Chen et al. [65] and Chang et al. [66] have reported that E1A causes downregulation of HSPA5 and HER2/neu expression and is positively associated with tumor metastasis. Taken together with these reports, oAd/shErbB3-mediated ErbB3 downregulation is predicted to cause multimodal anticancer effects, and it could be an interesting combination therapy candidate for ErbB2-targeted therapies to address the ErbB2/ErbB3 heterodimerization-induced drug resistance in the future. 

There have been several studies showing clear advantages of an oncolytic virus vector combining with RNAi-mediated oncogene silencing [64]. The oncolytic adenovirus expressing shIL-8 or c-met shows potent antitumor effects in a tumor xenograft model [35,36]. Mortalin-targeting oncolytic adenovirus was also shown to cause apoptosis in MCF7 cells [67]. Dual silencing of Bcl-2 and survivin by an oHSV-1 vector demonstrates antitumor efficacy in cancer cells [65]. Further, there are trials focusing on developing and establishing this platform more effectively by optimizing the structure and format of RNAi [66,68]. The reasons for these studies continuously conducted are as follows: (1) this therapeutic system can overcome the limitation of synthetic siRNAs exerting moderate therapeutic efficacy, which is mainly due to short half-life in vivo and (2) it can induce cancer specific amplification of RNAi where the replicating vector could infect and replicate, which eventually can lead to therapeutic potential without any safety issue. Taken together, oncolytic adenovirus combining with RNAi will be used as a new platform for tumor therapy in the future.

## 4. Materials and Methods

### 4.1. Cell Lines and Cell Culture 

All cell culture media were supplemented with 10% fetal bovine serum (FBS; GIBCO-BRL, Grand Island, NY, USA) and 1% of penicillin-streptomycin solution (100 U/mL). A human embryonic kidney cell line expressing the Ad E1 region (293A), lung cancer cell line (A549), and breast cancer cell lines (BT474, MDA-MB-231, SK-BR-3, and T47D) were purchased from the American Type Culture Collection (ATCC, Manassas, VA, USA). Mortalin-overexpressing MCF-7 (MCF-7/Mot) was generated by retroviral vector as described previously [69,70]. All cells were cultured in Dulbecco’s Modified Eagle’s Medium (DMEM; GIBCO-BRL) and maintained at 37 °C in a humidified atmosphere containing 5% CO_2_.

### 4.2. Construction and Generation of an Ad-Expressing ErbB3-Specific shRNA 

To generate adenoviruses expressing shErbB3 in the Ad E3 region, we first constructed a pSP72-E3 Ad E3 shuttle vector expressing shErbB3. The sequence of shErbB3 was derived from a previous study utilizing siRNA-targeting ErbB3 [29]. The DNA fragment targeting position 245-265 of human ErbB3 was generated by annealing the following two complementary oligodeoxynucleotides: the sense oligonucleotide, 5′- gatcccAACCAATACCAGACACTGTACttcaagagaGTACAGTGTCTGGTATTGGTTttttttggaaa -3′, and its cognate antisense oligonucleotide, 5′- agcttttccaaaaaaAACCAATACCAGACACTGTACtctcttgaaGTACAGTGTCTGGTATTGGTT gg -3′. The 21-nucleotide sequences targeting ErbB3 are indicated in uppercase letters, whereas the 9-nucleotide hairpin and sequences necessary for directional cloning are depicted in lowercase letters. The annealed fragment was then subcloned into the pSP72-E3 Ad shuttle vector [71], generating pSP72-E3/shErbB3. The newly constructed pSP72-E3/shErbB3 shuttle vector was linearized with *Xmn*I digestion. pdl-∆E1, a replication-incompetent Ad total vector, or pH5CmTERT-Ad, a total vector expressing the adenovirus *E1*A gene under the control of the H5CmTERT promoter [72], were linearized with *Spe*I digestion. The linearized pSP72-E3/shErbB3 E3 shuttle vector was then co-transformed into *Escherichia coli* BJ5183 along with the *Spe*I-digested pdl-∆E1 or pH5CmTERT-Ad for homologous recombination, resulting in the pdl-∆E1/shErbB3 Ad vector or pH5CmTERT/shErbB3 Ad vector. To produce the corresponding Ads and control Ads, purified plasmids were digested with *Pac*I and transfected into 293A cells to generate dl-∆E1 (dAd), dl-∆E1/shErbB3 (dAd/shErbB3), H5CmTERT (oAd), or H5CmTERT/shErbB3 (oAd/shErbB3). The replication-incompetent Ads were propagated in the 293A cell, whereas the replication-competent Ads were propagated in the A549 cells. The purification and titration of the viruses were performed in the same manner as described previously [73].

### 4.3. ErbB3 ELISA

The breast cancer cells (3 × 10^5^/well) were seeded on six-well plates with medium containing 10% FBS. The cells were then transduced with dAd or dAd/shErbB3 at various multiplicity of infection (MOI). At 72 h post-transduction, ErbB3 expression level was quantified in cell lysates using the human ErbB3/HER3 DuoSet ELISA kit (R&D Systems, Minneapolis, MN, USA) following the manufacturer’s recommendations. Serial dilutions of a known concentration of purified recombinant human ErbB3 protein were used to establish a standard curve.

### 4.4. Colony Formation Assay

MDA-MB-231 cells were infected with adenoviruses at an MOI of 100. At 24 h post-infection, the cells were harvested with trypsinization and seeded into 6-well plates in triplicates at a density of 1 × 10^5^ cells/well. After 14 days of incubation, the colony formation assay was carried out as previously reported [74]. 

### 4.5. MTT Assay

Breast cancer cells (MDA-MB-231, SK-BR-3, T47D, and BT474) were seeded (2 × 10^4^ cells/well) in 48-well plates and transduced with either dAd or dAd/shErbB3 (MOI: 50–500). At 0, 2, 4, and 6 days post-transduction, 250 μL of 3-(4,5-dimethylthiazol-2-yl)-2,5-diphenyl-tetrazolium bromide (MTT; Sigma Chemical, St. Louis, MO) in phosphate-buffered saline (PBS; 2 mg/mL) was added to each well. After 4 h incubation at 37 °C, the supernatant was discarded, and formazan was dissolved with 1 mL of dimethyl sulfoxide (DMSO). Absorbance was measured on a microplate reader at 540 nm. To measure cell viability after infection with oncolytic Ads, BT474 cells were seeded and infected with 2 MOI of oAd or oAd/shErbB3. MTT assay was performed every day from day 0 to 4 days post-infection as described above.

### 4.6. Cell Cycle Analysis

Cells were transduced with dAd or dAd/shErbB3 at an MOI of 50. At 48 or 72 h post- transduction, cells were detached with trypsinization, washed with PBS, and fixed in 70% (*v*/*v*) ethanol. For assessment of DNA contents, cells were stained with propidium iodide (PI) (50 μg/mL with 0.5 mg/mL RNase in PBS, pH 7.4) in the dark for 30 min, and then monitored by a fluorescence-activated cell sorter (FACS) using a BD LSR II (Becton Dickinson, San Jose, CA, USA). The data from 10,000 cells were collected and analyzed using a CellQuest program (Becton Dickinson). The FACS data were further analyzed using ModFit LT software (Verity Software House Inc., Topsham, ME, USA) to calculate the fraction of cells in subG1, G1, S and G2 phases.

### 4.7. Hoechst Staining

In order to detect chromatin condensation and nuclear fragmentation, nuclei were stained with Hoechst 33258 (Sigma). MDA-MB-231 (3 × 10^4^) cells were plated on either a 4- or 8-well chamber slide. After the cells were attached to the surface, they were transduced with dAd or dAd/shErbB3 at an MOI of 100. Cells treated with 1 μM of CPT were used as a positive control. At 72 h post-treatment, cells were fixed with 4% paraformaldehyde, and then stained with Hoechst 33258 (0.5 μg/mL) for 20 min at room temperature. 

### 4.8. Immunoblotting Analysis

At 48 h post treatment with replication-deficient Ad (dAd) or oncolytic Ad (oAd or oAd/shErbB3), BT474 cells were harvested and lysed in RIPA buffer (Elpis biotech, Seoul, Korea). The precleared lysates were separated by 12% sodium dodecyl sulfate-polyacrylamide gel electrophoresis (SDS-PAGE) and transferred to a polyvinylidene difluoride membrane. The membranes were blocked at room temperature for 1 h, followed by incubation at room temperature for another 1 h with the following antibodies: anti-ErbB2, anti-ErbB3, or anti-E1A. All antibodies, except anti-E1A, were purchased from Cell Signaling Technology (Beverly, MA, USA), and anti-E1A was purchased from Santa Cruz Biotechnology (Santa Cruz, CA, USA). All membranes were subsequently incubated at room temperature for 1 h with horseradish peroxidase (HRP)-conjugated secondary antibodies, goat anti-rabbit IgG HRP or goat anti-mouse IgG HRP (Cell Signaling Technology). Anti-β-actin (Cell Signaling Technology) was used as an internal loading control. Finally, the blots were developed using enhanced chemiluminescence (ECL) (Pierce, Rockford, IL, USA), imaged using the LAS4000 Luminescent Image Analyzer (Fujifilm Corp., Tokyo, Japan). 

### 4.9. The Antitumor Effect in a Human Breast Cancer Xenograft Model

Tumors were implanted subcutaneously into the right abdomen of 5- to 6-week-old female nude mice (Charles River Japan, Inc., Yokohama, Japan) by inoculating MDA-MB-231 (2 × 10^7^) or MCF-7/Mot (2 × 10^7^) cells in 50 μL of Hank’s balanced salt solution (GIBCO-BRL). When the average tumor volume reached 150–200 mm^3^, PBS, dAd (2 × 10^10^ VP), or dAd/shErbB (2 × 10^10^ VP) were administered to MDA-MB-231 tumor-bearing mice intratumorally on day 0, 2, 4, and 6. MCF-7/Mot tumor-bearing mice were treated with PBS, oAd (2 × 10^10^ VP), or oAd/shErbB (2 × 10^10^ VP) via intratumoral administration on day 0, 2, and 4. MDA-MB-231 tumor-bearing mice were treated with 1 × 10^10^ or 2 × 10^10^ VP of oAd or oAd/shErbB, along with PBS control, via intratumoral administration on day 0, 2, and 4. The day of first injection was designated as day 0 of treatment. Tumor volume was measured every alternative day by measuring the length (L) and width (W) of the tumor using a digital caliper. The tumor volume was calculated using the following formula: volume = 0.523L(W)^2^. The survival rate was analyzed by monitoring the tumor growth-related events (mice were considered dead when tumor size exceeded 3000 mm^3^). All mice were maintained in a laminar airflow cabinet under specific pathogen-free conditions. All the facilities have been approved by the Association and Accreditation of Laboratory Animal Care, and all animal-related experiments were conducted under the institutional guidelines established by the University of Hanyang Institutional Animal Care and Use Committee, Seoul.

### 4.10. Histology and Immunohistochemistry

For histological analyses, tumor tissues were embedded in paraffin, and sectioned at a 3 μm thickness. Representative sections were stained with hematoxylin and eosin (H&E), and then examined under light microscope. For immunohistochemical staining, the tumor slices were deparaffinized in xylene, and then hydrated through graded alcohols. The tumor slices were blocked with Protein Block Serum-Free Solution (DAKO, Glostrup, Denmark), followed by staining with anti-ErbB3 (Cell Signaling Technology) or monoclonal mouse anti-proliferating cell nuclear antigen (PCNA; Santa Cruz Biotechnology) as primary antibodies, which then reacted with the secondary antibody (DAKO REAL^TM^ EnVision/HRP, Rabbit/Mouse (END); DAKO). All tumor slices were counterstained with Meyer’s hematoxylin (Sigma). A terminal deoxynucleotidyl transferase dUTP nick end labeling (TUNEL) assay was performed as described previously [75].

### 4.11. Statistical Analysis

All data are expressed as mean ± standard deviation (SD) or standard error (SE). All statistical analyses were carried out by the Student’s t-test and one-way ANOVA (SPSS 13.0 software; SPSS, Chicago, IL, USA). The p values less than 0.0.5 were considered statistically significant.

## Data Availability

All datasets used and/or analyzed during the current study are available in the manuscript and supplementary information files.

## Supplementary Materials

The following supporting information can be downloaded at: https://www.mdpi.com/article/10.3390/ijms23137127/s1.
