# Supplementary material for: ErbB3-Targeting Oncolytic Adenovirus Causes Potent Tumor Suppression by Induction of Apoptosis in Cancer Cells
Source: Int J Mol Sci. 2022 Jun 27;23(13):7127. doi: 10.3390/ijms23137127 (PMC9266575; doi:10.3390/ijms23137127)
Supplement: Supplementary file 1 [file ijms-23-07127-s001.zip › ijms-1740071-supplementary.pdf]

International Journal of *Molecular Sciences*

MDPI

Article

ErB3-targeting oncolytic adenovirus causes potent tumor suppression by induction of apoptosis in cancer cells

Bo-Kyeong Jung[1,2], Young Jun Kim[1], JinWoo Hong[1,2], Han-Gyu Chang[1], A-Rum Yoon[1,3,4], and Chae-Ok Yun[1,2,3,4*]

1 Department of Bioengineering, College of Engineering, Hanyang University, 222 Wangsimni-ro, Seongdong-gu, Seoul 04763, Republic of Korea; yeesule@hanyang.ac.kr (B-K.J.); mark95@hanyang.ac.kr (Y.J.K.); jhong803@gmail.com (J.H.); charley26@hanyang.ac.kr (H.-G.C.); ayoon@hanyang.ac.kr (A-R.Y.); chaeok@hanyang.ac.kr (C-O.Y.)

2 GeneMedicine Co., Ltd., Seoul, 04763, Republic of Korea

3 Institute of Nano Science and Technology (INST), Hanyang University, 222 Wangsimni-ro, Seongdong-gu, Seoul 04763, Republic of Korea

4 Hanyang Institute of Bioscience and Biotechnology (HY-IBB), Hanyang University, 222 Wangsimni-ro, Seongdong-gu, Seoul 04763, Republic of Korea

* Correspondence: chaeok@hanyang.ac.kr (C-O.Y.)

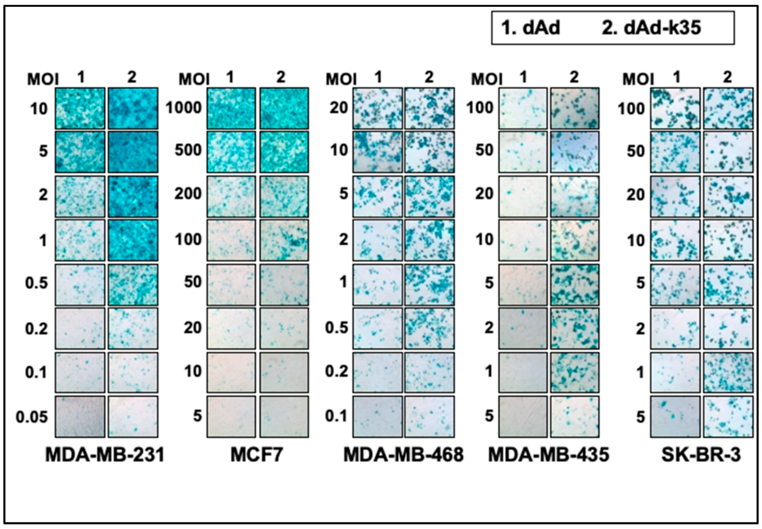

Supplementary Figure S1. Enhanced transduction efficiency of dAd-k35-LacZ. Human breast cancer cells (MDA-MB-231, MCF7, MDA-MB-468, MDA-MB-435, and SK-BR-3) were transduced with dAd-LacZ (lane 1) or dAd-k35-LacZ (lane 2) with various amount of virus (0.05 - 1000 MOI). At 48 h post-transduction, cells transduced with dAd-LacZ or dAd-k35-dAd-LacZ were stained with X-gal for analyzing the β-galactosidase.

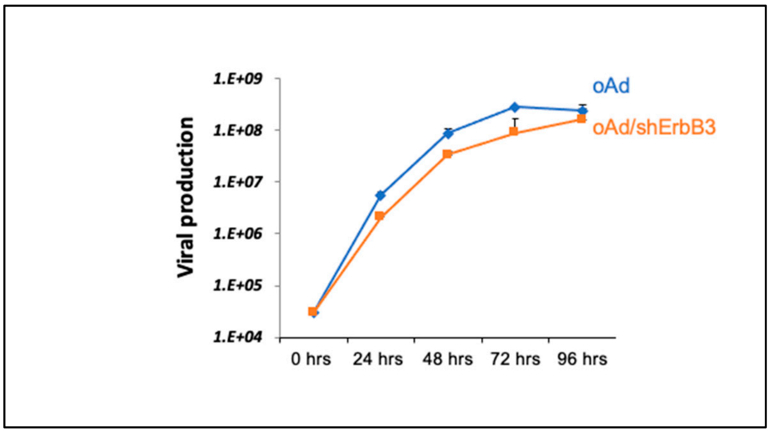

Supplementary Figure S2. Viral replication of oAd and oAd/shErbB3. MDA-MB-231 cells seeded in a 12-well plate were infected at MOI of 0.1 with oAd or oAd/shErbB3. At 0, 24, 48, 72, or 96 h post infection, viral genome copy numbers were measured by Q-PCR.

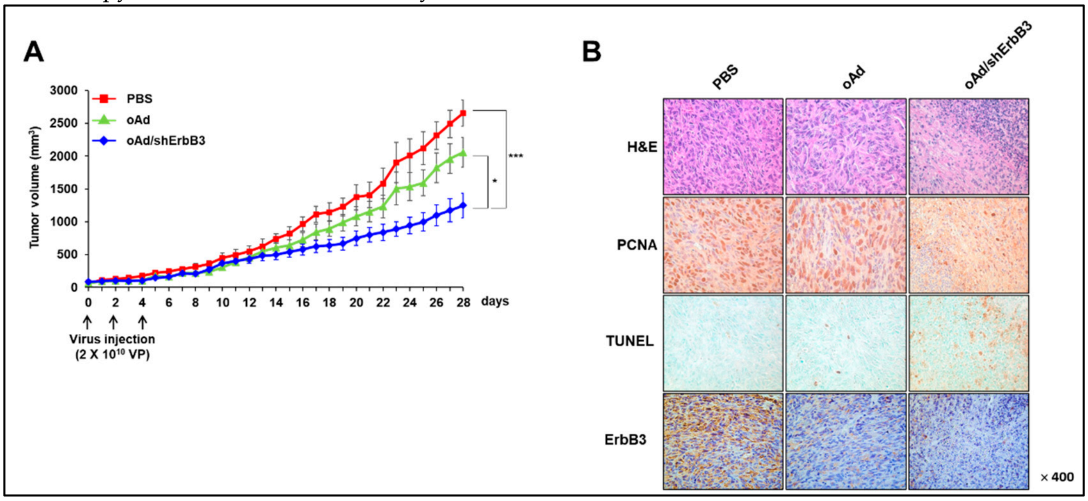

Supplementary Figure S3. Tumor growth inhibition by shErbB3-expressing oncolytic Ad. MCF-7/Mot tumor-bearing mice were injected with 2 x 10^{10} VP of either oAd or oAd/shErbB3 along with PBS control (Q2D x 3). *p<0.01 or ***p<0.001. **(A)** Tumor volume was measured every other day. Data represent as mean ± SE (n = 5 to 8). **(B)** Histological and immunohistochemical analysis. Tumors treated with PBS, oAd, or oAd/shErbB3 were harvested and subjected to H & E staining and immunohistochemical staining for PCNA, TUNEL, and ErbB3, respectively.
